# Zigzag Barbed Polydioxanone Thread Implantation and Evaluation Using Polydimethylsiloxane Model to Simulate Thread Migration in Tissue

**DOI:** 10.3390/polym16131785

**Published:** 2024-06-24

**Authors:** Chia-Hsien Hsieh, Yi-Xin Liu, Pei-Yu Chen, Hsu-Wei Fang

**Affiliations:** 1 Department of Chemical Engineering and Biotechnology, National Taipei University of Technology, No. 1, Sec. 3, Zhongxiao E. Rd., Taipei 10608, Taiwan; t106679007@ntut.org.tw (C.-H.H.); dr.evieliu@gmail.com (Y.-X.L.); peiyu.chen@biotegy.com (P.-Y.C.); 2 High-Value Biomaterials Research and Commercialization Center, National Taipei University of Technology, No. 1, Sec. 3, Zhongxiao E. Rd., Taipei 10608, Taiwan; 3 Institute of Biomedical Engineering and Nanomedicine, National Health Research Institutes, No. 35, Keyan Road, Zhunan Town 35053, Miaoli County, Taiwan

**Keywords:** thread-implantation method, polydimethylsiloxane model, zigzag barbed polydioxanone

## Abstract

Facial lifting with polydioxanone barbed threads has been widely used in aesthetic treatment for years. However, gravity resists the thread and continuously pulls the face downward. This study aims to determine methods to lift the skin more efficiently with longer longevity. The quality of the thread is important and is defined by the pulling and pullout strengths. Moreover, the method of using threads is also important. We compared five thread-implantation techniques and six angles for the V-shaped implantation methods using a polydimethylsiloxane model to simulate thread migration in tissues. The results of the simulated thread-lift techniques can provide valuable information for physicians, enabling a more precise design of facelift surgery techniques.

## 1. Introduction

Facial aging is a dynamic process involving dramatic changes in different facial structures, including the skin, soft tissues, and facial skeleton. Thread lifting is a cosmetic procedure that offers a minimally invasive alternative to facelift surgery. Thread lifts claim to tighten the skin by inserting medical-grade thread material into the face and “pulling” the skin up by tightening the thread [1,2]. This technique has been used since the 1990s, and innovations in materials used for thread lifts have increased in international popularity [3,4,5,6]. Thread materials with various shapes and surface characteristics are available on the market [7,8]. Moreover, thread technologies, techniques, and procedures are evolving [9,10,11]. Improved patient experience and an increase in the number of peer-reviewed publications contributed to the body of evidence and helped improve safety and efficacy. Patient selection is of the utmost importance, along with patient communication and understanding of the procedure, before- and after-care and management of patient expectations determine the fine line between patient satisfaction and dissatisfaction. In particular, patients should undergo a comprehensive consultation with the discussion of the complete information regarding preoperative care, the procedure, expectations, pretreatment and posttreatment care, recovery time, and possible need for multidisciplinary treatment. It is important to emphasize to the patients that this procedure is different from a surgical facelift, which results in a quick “lift” with no downtime.

Polydioxanone (PDO) thread is an absorbable polymer composed of paradioxanone monomer and degrades into water and carbon dioxide in the body [12]. PDO threads have been used as sutures. After processing, either by cutting or molding, the threads proved useful for minimally invasive face lifting. This was achieved by inserting a cannula into the face, placing the thread into the cannula, and then removing the cannula to leave the thread inside the skin. The results improved as the number of threads used increased. The thread not only lifts the skin physically but also stimulates collagen biosynthesis, vascular neogenesis, and fat reduction [13].

The lifting efficiency of this method can be influenced by the quality of the threads and the threading techniques [4,14,15]. In particular, the technique for using the threads in a straightforward manner or creating an undetermined loop on the skin to achieve a better and longer lifting effect has been debated [10]. Therefore, it will be critical to establish an in vitro testing method that allows evaluation of different lifting techniques consistently and individual differences of patients can be eliminated.

In this study, we designed an experiment to quantify the pull-out strength in vitro using different methods [16]. We used a thread with a zigzag barbed construction containing multiple barbs, a planar structure, and multiple grooves of specific sizes. We used an in vitro tensile testing model developed in a previous study [17] to simulate commonly used surgical techniques and investigate the relationship between different suture placement methods, surgical tension, and displacement. Synthetic polydimethylsiloxane (PDMS) was used to mimic the human skin, and the tensile strength of the barbed sutures was evaluated using a universal material testing machine. The effects of different suture placement methods, including single, double, V-shaped, and U-turn-shaped, on the distance of the facial tissue displacement caused by applied tension were studied [18,19,20]. In the force-displacement graphic profile of the V- and U-turn shape placements, the pull force continuously rises after the first peak point falls. Therefore, we conducted further tests to verify the influence of the V-shaped surgical threading angle (0°–30°) on the holding strength of the tissue and the relationship between tension and topology.

## 2. Materials and Methods

### 2.1. Barbed Thread Sample

Meteora Lifting Premium (Diamond Biotechnology Co., Ltd., Taipei, Taiwan) with a size of USP 1 was used as the zigzag barbed thread. The Meteora lifting thread consists of violet-dyed polyester and synthetic absorbable PDO. The pigment of the violet dye (21 CFR §74.3602, D&C Violet No. 2) was sterilized using ethylene oxide and degraded in tissues over time. The Meteora lifting thread has bidirectional zigzag barbs along its long axis. The opposing barbs on the thread surface were embedded in the tissue after the surgeon precisely placed the thread within the tissue. The average tensile strength of the wire measured by a universal material testing machine is 5.2 ± 0.03 kgf (51 ± 0.3 N).

### 2.2. Polydimethylsiloxane (PDMS) Specimen Preparation

PDMS specimens were prepared according to the manufacturer’s instructions (SLY-GARD 184 Silicone Elastomer; Dow Corning; Midland, MI, USA). Briefly, the base reagent and curing reagent (30:1) were thoroughly mixed for 20 min and then poured into a 90 mm Petri dish as a mold to obtain a disk with a radius of 45 mm and thickness of 10 mm (~50 g). The PDMS specimen was then cured for 2 h in an oven at 70 °C for use.

### 2.3. Barbed Suture Implantation

**Figure 1:**
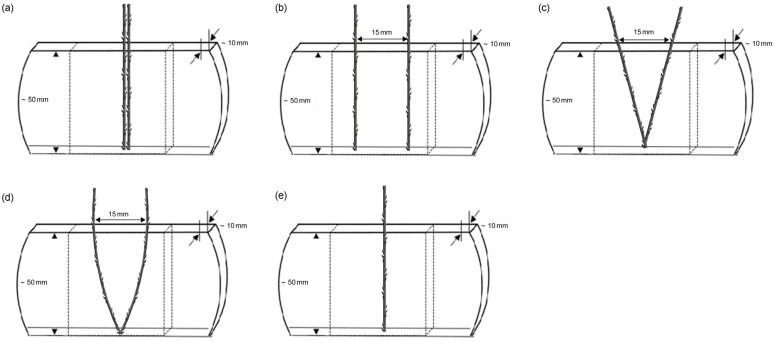
Five different barbed suture implantations: (**a**) Straight-line double strand, (**b**) two parallel lines, (**c**) V-shaped, (**d**) U-shaped, and (**e**) single lines. The test samples were inserted into PDMS to a fixed depth of 50 mm.

For the pull-out test, a cannula (blunt needle) with a zigzag barbed thread was inserted into the PDMS specimen to guide the thread and then removed from the PDMS, leaving the barbed thread inside the specimen. The threads were inserted into PDMS using different suture placement methods, namely, straight-line double strand (Figure 1a), parallel double lines (Figure 1b), and V-shaped (Figure 1c), U-shaped (Figure 1d), and single (Figure 1e) lines. The specimens were tested using five replicates for each experiment.

### 2.4. V-Shaped Implantation at Different Angles

**Figure 2:**
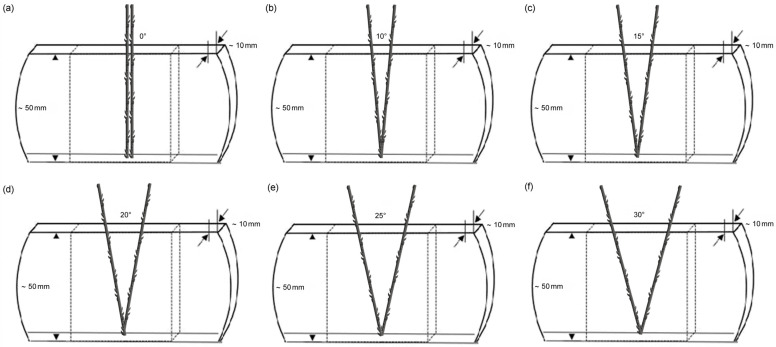
Six angles of the V-shaped threading implantation: (**a**) 0°, (**b**) 10°, (**c**) 15°, (**d**) 20°, (**e**) 25°, and (**f**) 30°. The test samples were inserted into PDMS at a fixed depth of 50 mm.

In clinical practice, the surgical threading spread angle is typically 15°–30°. The relationship between tension and displacement at different angles (0°–30°) at intervals of 5° was simulated. The test samples were inserted into PDMS using methods similar to that described in 2.3 with different V-shaped angles. Six groups of V-shaped angle samples at angles of 5°, 10°, 15°, 20°, 25°, and 30° were prepared (Figure 2). The specimens were tested in five replicates for each experimental group.

### 2.5. Pull-Out Test

The pull-out test was previously described [16]. In this study, the PDMS specimen inserted with the barbed thread was fixed in a universal testing machine (YM-H35, Yang Yi Technology Co., Ltd., Tainan, Taiwan). The zigzag barbed thread sample was clamped to the upper jaw, whereas the specimen was clamped to the lower jaw of the testing machine; the distance between the two clamps was 100 mm. The upper clamp was driven at a speed of 100 mm/min. All data (e.g., force and displacement) were recorded using QCTech3_A2 software. The changes in the barb image before and after the test were detected using a scanning electron microscope (SEM; Hitachi TM4000, Tokyo, Japan).

### 2.6. Data Analysis

Five samples in each group were investigated. We selected four to five samples with high repetition rates to calculate the slope, intercept, and trend line.

## 3. Results

**Figure 3:**
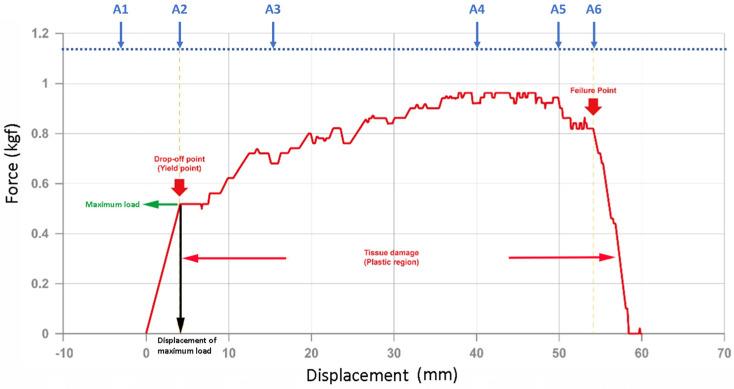
Representative result of the pull-out test. The force increases during the pull-out test when the barbs grip the specimen. When the barbs failed, the force decreased (red arrow); the force at this point is defined as the “maximum load” and the moving distance is the “displacement of maximum load”. A1–A6 are the places pointed by the arrows respectively. The schematic photos of each time point are shown in Figure 4.

**Figure 4:**
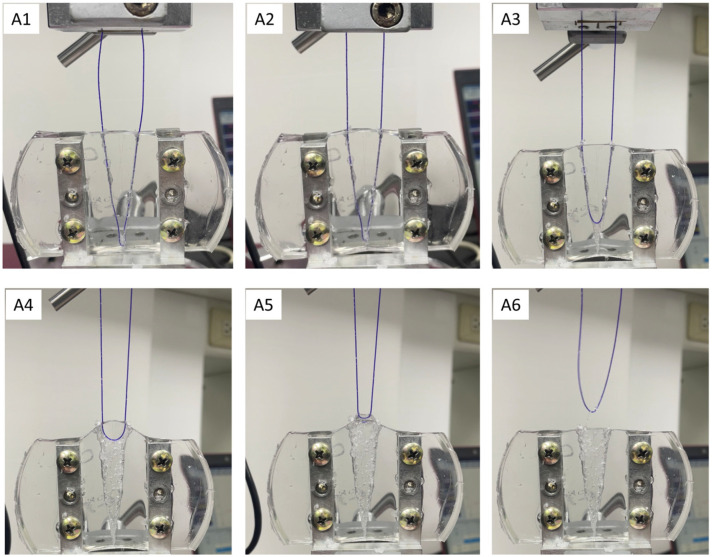
These are representative photos of the pull-out test process at marked points A1–A6 in Figure 3.

**Figure 5:**
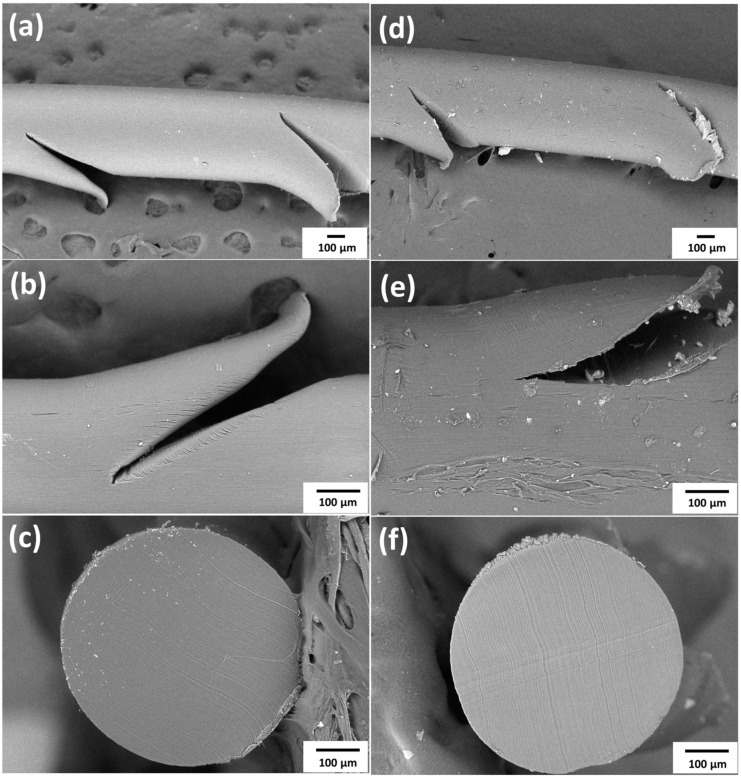
SEM images before and after the V-shaped pull-out test, before the test (**a**) side, (**b**) barb, (**c**) cross-section; after test (**d**) side, (**e**) barb, (**f**) cross-section.

When the zigzag barbed thread was gradually pulled from the specimen during the pull-out test, the barbs first grasped the specimen, which increased force during the test period, indicating the increasing holding strength of the barbs (Figure 3). Photos A1–A6 of the V-shaped experimental process were used as a representative reference in Figure 3 (Figure 4). In this region, the forces and displacements are linearly related, and the slope of the curve is called the “tensile modulus” of the tissue. Subsequently, the strength profile greatly decreased, which is defined as the “drop-off point” or “yield point” (Figure 3, red arrow). The slope of the profile changes and the plastic region begins. After the “drop-off point,” the tissue begins to experience destructive changes as the zigzag barbs start to detach and slip from the gripped site (Figure 3, “failure point”) [21,22]. The force gradually declined after the significant “failure point” as the entire suture was pulled out and the barbs lost their holding capacity. The drop-off point is a typical parameter reported for soft tissues under destructive testing. Additionally, the material structure before and after the pull-out test was observed using SEM, and the tangent opening at the barb was deformed, and more friction marks appeared on the wire surface after the pull-out test (Figure 5a,b,d,e). However, the wire body was not broken and there were no special changes inside (Figure 5c,d). The results revealed that the structure became loose after the test. Therefore, in this study, each thread was subjected to the pull-out test only once.

### 3.1. Pull-Out Strength of Zigzag Barbed Thread with Different Implantation Methods

**Figure 6:**
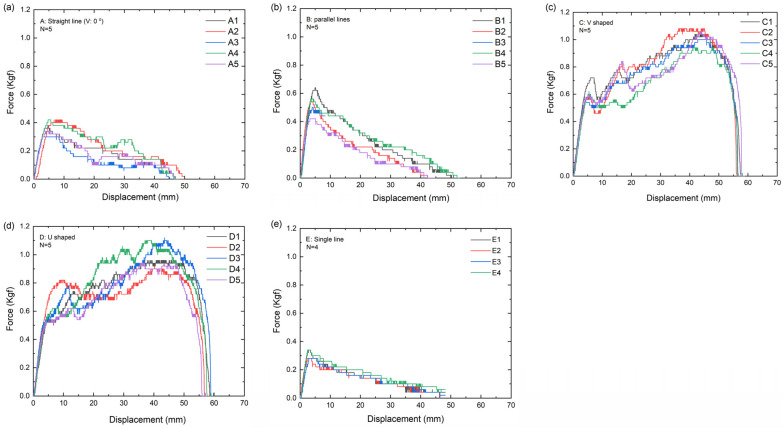
Pull-out force and displacement results of the five implementation methods: (**a**) straight-line double-strand, (**b**) two parallel lines, (**c**) V-shaped, (**d**) U-shaped, and (**e**) single line. The maximum load in the curve indicates the force that should be applied to start the thread moving from PDMS.

The displacements of the threads are shown in Figure 6. When the barbed thread first grasped the PDMS specimen, the force increased during the pull-out test, indicating the increased holding capacity of the barbs [16]. When the curve deviated from the trend line, the thread dehooked the PDMS. The first point on the curve represents the force required to cause the thread to start moving. As the force increases, the pull-out strength and the force of this point are defined as the “maximum load.” The highest maximum load was noted in group D, followed by groups C, B, A, and E. The curves decreased after the “maximum load” in groups A, B, and E, and increased in groups C and D.

The slope of the curve in Figure 6 indicates the holding capacity at a constant pull-out speed of 100 mm/min before the thread began moving. Group B has the highest average maximum holding capacity, followed by groups C, D, E, and A.

**Figure 7:**
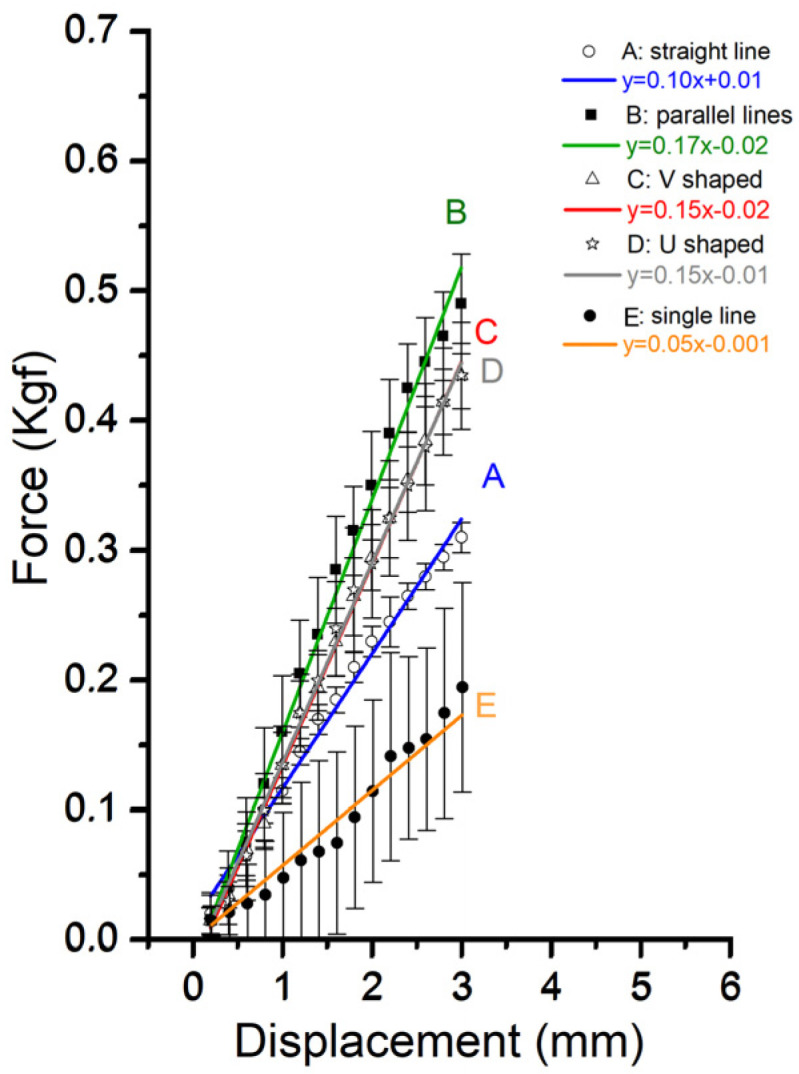
Force-displacement profile slopes of five different implantation threading methods: (A) straight-line double strand, (B) two parallel lines, (C) V-shaped, (D) U-shaped, and (E) single lines.

In this study, a trend line was fitted to the pre-unhooked data to calculate the slope and intercept. When the tension curve deviates from the trend line, it is considered as approaching unhooking. As shown in Table 1 and Figure 7, the data for n = 4 or 5 were used, and trend lines were constructed using the data, with an interval of 0.2 mm at a displacement of 0–3 mm. Before unhooking, the slopes of the lines intersecting the two lines in groups B, C, and D exceeded 0.15–0.17 for group B. Meanwhile, the slopes of groups A and E are 0.10 and 1.2, respectively.

### 3.2. Pull-Out Strength of Zigzag Barbed Thread for V-Shaped Implantation at Different Angles

**Figure 8:**
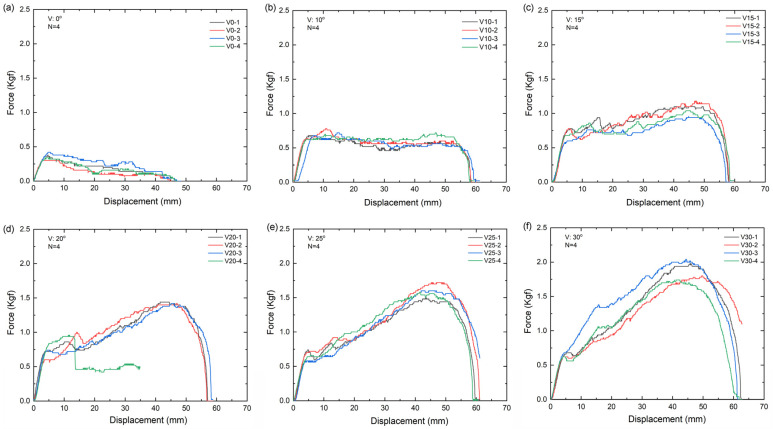
Force-displacement profile of V-shaped thread implantation at different angles: (**a**) 0°, (**b**) 10°, (**c**) 15°, (**d**) 20°, (**e**) 25°, and (**f**) 30°.

**Figure 9:**
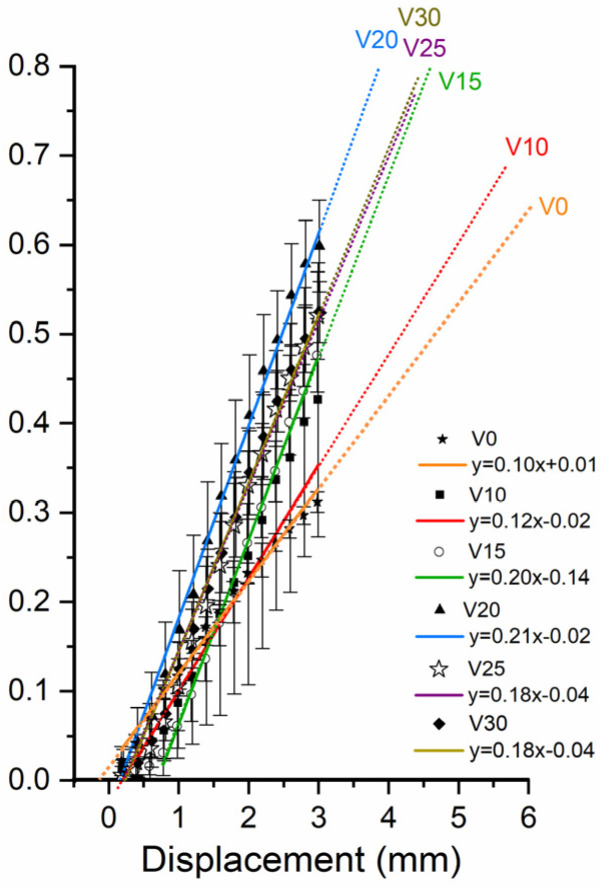
Force-displacement profile slopes of V-shaped implantation at different angle threading methods.

The force-displacement results at different angles are shown in Figure 8, and the slope and intercept as shown in Figure 9. The area under the force-displacement curve and accumulated energy required to slip and dispatch the barbed thread from the PDMS specimen are listed in Table 2. The highest accumulated energy was obtained at an angle of 30°, followed by 25°, 20°, 15°, 10°, and 0°. When the first peak is exceeded, the part is considered to be unhooked and the PDMS at the bottom is also damaged. This can be regarded as the destruction of the skin tissue during surgery.

When all the V-shaped angles exceeded 15°, the holding capacities increased significantly during the pull-out test process before the barbs failed, which can be regarded as the destruction of skin tissue. Skin is considered a nonlinear elastic material with low strain-rate sensitivity [23]. Most studies on the mechanical deformation of skin have focused on collagen, which is the main structural component of the dermis. Collagen deformation occurs at several distinct stages. Therefore, our study is based on the concept of nondestructive subcutaneous tissue, and the difference in these pull-out strengths was detected with the in vitro PDMS model, which can provide valuable information for physicians, enabling a more precise design for facelift surgery.

## 4. Discussion

The previously established pull-out strength test method was shown to offer objective information for barbed sutures that could be applied to facelifts [16]. For current experimental geometries, destructive and nondestructive testing could be conducted. In the nondestructive protocol, the tissue was tested with small strains or loads, and all changes induced in the tissue were reversible. In contrast, destructive protocols involved larger strains or loads that induced irreversible changes in the tissue. The most common nondestructive testing protocols were creep and stress relaxation. In the creep test, constant tensile stress was applied to the tissue, and the corresponding strain was measured as a function of displacement. Destructive testing is typically conducted for soft tissues only in a tension geometry. The mechanical behavior of the tissue can be deduced from the force-displacement curve.

PDMS is a biomaterial widely used in medical practice to simulate human body parts [24] owing to its low cost and ease of handling. In this study, consistent results were achieved using PDMS (30:1) as the specimen for the barb pull-out test (Figure 6 and Figure 8), which could be attributed to the homogeneity of the material. The in vitro pull-out tests noted a significant maximum load for the pull-out strength of barbed sutures with different implantation methods (Figure 6 and Figure 7). The highest maximum load was obtained in group D, followed by groups C, B, A, and E, respectively, indicating that the U-shaped threading implantation method requires the highest strength to resist moving, whereas the single-thread implantation method requires the lowest strength. The tension displacement lines of groups A, B, and E exhibited a peak triangle, whereas those of groups C and D were trapezoidal. This denotes the larger spread of the PDMS for the latter group, which can affect the total work due to the barbed thread movement. The exceeding peak represents the unhooked part and damaged PDMS at the bottom. During surgery, this can be regarded as the destruction of the skin tissue. Therefore, this study was based on the concept of nondestructive subcutaneous tissue analysis, in which only the pre-unhooking data were utilized as a reference. After the maximum load, the curves decreased in groups A, B, and E, whereas they increased in groups C and D. This denotes that the V- and U-shaped implantation methods can resist the pull-out strength even when the barbs were dehooked, which can be ascribed to larger grasps of the V- and U-shaped implementations between the threads.

**Figure 10:**
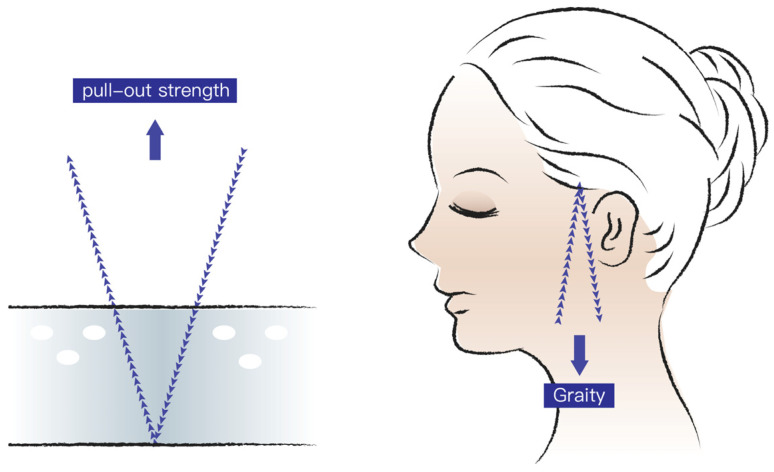
In vitro study creates a V-shaped pattern within the PDMS and pulls the thread upward. When applying the result clinically, the V shape is upside down, and gravity represents the pull-out strength. According to the in vitro study, this pattern makes the thread more resistant to gravity compared to the parallel pattern. The pull-out strength works as gravity for the face.

Strength is required to elongate the threads before they start moving. Group B obtained the highest elongation, followed by groups C, D, E, and A. This implies that the two-thread parallel-type implantation can facilitate larger elongation than the V- and U-shaped methods. A reasonable explanation is the parallel direction of the pull-out strength to the straight-line double-strand method, whereas the two parallel lines and V-shaped method are at an angle, which diminishes the total force of the pull-out direction. Thus, when thread lifting is performed clinically, a V- or U-shaped pattern should be used to prevent the thread from moving (Figure 10). This can also explain why there are higher satisfaction rates when the threads are inserted with a special technique to make the pattern look like a slash in the skin with the basis of a reverse V on the top [25]. The reverse V acts as a strong anchoring for the thread to pull up the skin and prevent it from sagging again. Additionally, with this method, we can prevent the thread from migrating. About the angel between both legs of the reverse V, although the study showed the maximum load is at a 30° angle, we still have to consider the tension balance of the skin because the wider the legs, the higher the unbalanced chance [26]. Theoretically speaking, the lasting time of the effect will be longer if the thread can provide the skin with a more powerful anchoring. Another key point for lasting is the grasping force of the barbs on the thread. The zigzag-shaped bard is thicker than the cutting barb and can give the thread stronger power to lift the sagging skin.

Different implantation angles were used for the V-shaped threading method to compare the resistances between both legs when a V-shaped pattern is applied. As the angle increases, the force needed to remove the thread from the PDMS specimen also increases (Figure 8). When the angle exceeded 20°, more PDMS spread and a greater pulling force was required to move the thread. A better grip is speculated when the suture is inserted on the human face with a large-angle threading method, requiring a larger external force. In contrast, a threading method with a small angle loosens [25,26,27,28]. Therefore, for facial thread lifting with a V-shaped implantation, an angle of 30° requires the greatest force to resist the extraction force from gravity.

No conclusive consensus is available concerning the number of threads and optimal location for placement of threads in thread lifting. However, a better understanding of the lifting technical vectors and considering cultural disparity may produce the desired results more successfully than the original placement [10].

Carcass stitching and lifting of flabby soft tissues (subcutaneous fat) of the middle area of the aging face using special suturing material should be followed by stable fixation in a new, aesthetically more advantageous position [29].

To anchor the cheek tissues with loops of non-absorbable sutures to the temporal fascia on the side of the head and avoid making visible scars, the “Suture Suspension Loops”, which was introduced in 1994, is used. Alternatively, or in combination, one can use specially designed anchoring threads to lift the midface tissues. These threads are anchored using 4–6 stab-incisions in a special pattern behind the temporal and side-burn hairline and one can easily see that traction on these tissues will eliminate the jowls and improve the corner of the mouth. This creates the classical “ogee” cheek contour [30]. One of the major advantages of this procedure is that the anchoring area can be marked with silicon or even metallic rings, and then at a later date, when the tissues start to sag again, they can be tightened via a small operation in the temporal area without any signs of surgical intervention in the face. This novel idea is derived from the anchoring system for the “Silhouette” suture lift, which is anchored onto the same anchoring system as the suture loops. The inferior part of the loop curves sufficiently to catch a good volume of malar fat and then goes straight up to the temple anchor point.

The “APTOS THREAD” method, with a technically suitable operation performed, is the most minimally invasive and at the same time rather effective. However, several reasons, such as underestimation of the issues related to specialists’ training and flooding of the market with poor-quality copies of the threads, have led to the appearance of a great number of inefficient outcomes in patients and even complications [31].

This study aimed to develop an in vitro evaluation method using a PDMS specimen to determine the lifting/pull-out strength of a suture, specifically the barbed suture, with simulation of pre-clinical efficacy and safety of the facelift results.

At present, there is no single percutaneous thread lift device and procedure that is regarded as the most effective [32]; moreover, there is still no consensus on the number of threads to be used or how to best position them, and little data are available to guide insertion techniques and material selection [33,34].

Traditional surgical and nonsurgical techniques for facial rejuvenation remain the golden standards for rejuvenation [35]. Although suspending the ptotic facial tissues like a marionette appears simple, more profound knowledge concerning the anatomic and physiologic basis of aging argues for the need for a surgical approach to redistribute the different layers and components by standard open or endoscopic facelifts.

The thread lift procedure has been popularized over the past 20 years. Several techniques have been developed to help meet patient expectations and improve patient satisfaction. The primary focus should be the areas of patient concern, the physician’s assessment of the physical problem, and the selection of the most appropriate technique for the patient. This study provides valuable information for physicians, enabling a more precise design of facelift surgery techniques.

## 5. Conclusions

Skin sagging is a major problem for individuals seeking facial rejuvenation. Surgical facial lifting or rhytidectomy has been performed for more than 100 years. However, such procedures have short-term sustainability of the outcomes.

In our study, V- and U-shaped implantation methods achieved maximum displacement distances of 2 mm at a tension force of ~0.3 kgf. In contrast, the single-strand method achieved the same distance as that of the V- and U-shaped samples with a tension force of less than 0.3 kgf. Thus, the V- and U-shaped methods required the highest strength to resist movement, whereas the single-thread method required the lowest strength. Furthermore, the V- and U-shaped implementation resisted the pull-out strength even when the barbs were unhooked, which can be ascribed to their larger tissue volume surrounded by the double thread lines.

For different angles in the V-shaped implantation, as the angle increased, the force needed to remove the thread from PDMS also increased. An angle of 30° between the bilateral legs required the greatest strength to resist the extraction force from gravity.

The results of the simulated thread-lift techniques provide valuable information for physicians, enabling a more precise design of facelift surgery techniques. More importantly, the longevity of the results can be improved using this approach.

## Figures and Tables

**Table 1:** Pull-out strength test results with different implantation methods.

Group	Model	Maximum Load (kgf)					Mean	Standard Deviation	Slope
A	Straight line double strand	0.38	0.42	0.30	0.42	0.34	0.37	0.05	0.10
B	Two parallel lines	0.64	0.54	0.50	0.56	0.42	0.53	0.08	0.17
C	V-shaped	1.04	1.08	1.00	0.94	1.06	1.02	0.06	0.15
D	U-shaped	0.96	0.92	1.12	1.10	0.94	1.01	0.09	0.15
E	Single line	0.38	0.42	0.34	0.42	-	0.39	0.04	0.12

**Table 2:** Pull-out strength at different V-shaped implantation angles.

Angle	Maximum Load (kgf)				Mean	Standard Deviation	Slope	Accumulated Energy (kgf × mm)
0	0.38	0.30	0.34	0.42	0.37	0.05	0.10	8.83
10	0.68	0.78	0.72	0.72	0.72	0.04	0.12	30.99
15	1.10	1.18	0.96	1.04	1.07	0.09	0.20	50.23
20	1.44	1.42	1.42	0.96	1.31	0.23	0.21	57.92
25	1.50	1.72	1.60	1.58	1.60	0.09	0.18	61.90
30	1.98	1.80	2.04	1.74	1.89	0.14	0.18	80.01

## Data Availability

The data presented in this study are available on request from the corresponding author due to privacy.
